# Results of Recent Remarkable Experiments with Young Dogs1From the Philadelphia Times.

**Published:** 1896-08

**Authors:** 


On to Buffalo!

---

### RESULTS OF RECENT REMARKABLE EXPERIMENTS WITH YOUNG DOGS.[1]

THE results of some remarkable experiments concerning the relations of the structures and functions of the mind have recently been announced by Professor Elmer Gates, a physiologist, who has for several years been making elaborate studies both in Washington and Philadelphia.

By training pet animals, especially dogs, to perform various difficult tricks, Professor Gates developed many strange abnormalities in the gray structures of their brains. In this manner he has not only located the exact parts of the brain in which even the most intricate emotions originate, but has convinced himself of a method by which the brain of man may be built entirely anew. His laboratory is one of the most interesting institutions of the kind in the world. Various instruments have been erected in it for aiding the professor in his peculiar training. Some of these work by electricity, a slight shock from a battery giving such a gentle reminder as is generally administered by the whip.

In one experiment he trained dogs so skilfully that they were able to distinguish colors, one from another, and associate them

[1] From the Philadelphia Times.

with action. Seven little shepherd puppies were kept in a totally darkened room from the minute they were born until they became nine months old. So careful was the professor lest even a spark should enter this room that he placed three different doors in the approach to it. A second group of little shepherd dogs of the same age were allowed to lead a normal life. A third group of the same age and stock were put through an interesting course of sight-training. To accomplish this the floor of the hall leading to one room of the laboratory was covered with square metallic plates, separated each from the other by insulation. These, with the exception of a few, were connected with the current of a strong electric induction-coil. Those not connected were irregularly scattered and painted a like color. The others were painted in various other shades, giving the whole floor a checkered appearance. At first when the dogs followed the professor to his laboratory they had their feet uncomfortably tickled, not knowing the proper combination. In a few months, however, after being trained a number of hours each day they gradually learned the proper plates, no matter how their positions were changed. Within five months the professor had perfected this unique education to his satisfaction.

Another interesting method of developing the color bumps was by feeding the same dogs meat placed under different colored pans, which had to be turned over with their noses before the food could be discovered. Each pan was rubbed with meat on the outside that they might not be led to the proper pan by smell. Each was shown that the yellow pans only contained the meat. For several weeks, however, they continued to turn over each pan indiscriminately until finding the dinner. After six weeks the proper places were found the first time, no matter in what manner their positions were altered. By placing the food under pans of other colors they soon became able to discriminate between seven different shades of red, several of green, and of still others.

The three groups of puppies when nine months old were chloroformed and their brains and spinal cords studied under powerful microscopes and by applying various chemical tests. Those kept in a dark room all their lives showed a total absence of development in the portion of the gray exterior covering, or cortex, which Professor Gates previously knew to be occupied by the "seeing area." This was totally lacking in cells. The group which had led a normal dog's life had much greater development in this "bump of sight," as the common phrenologist would call it. The cortex was thicker, more thickly supplied with arteries and veins. It was more gray in color, and contained cells. But the seeing area of the dogs highly trained showed a wonderful development. The cortex was abnormally thick and exceeded those of the normal dogs in the number of cells and bloodvessels. The cells were much more complex in form. In short, in that portion of the brain where originates the sense of sight the trained dogs were found to be twenty-five times as complex as the group which had led a usual life. It therefore seems very apparent that similar training applied to human beings deficient in brains might accomplish untold wonders in a few years, if only nine months would improve a separate portion of a dog's mind twenty-fivefold.

Professor Gates has obtained equally wonderful results not only with dogs but with other animals. He grouped some of these so that part were deprived of hearing, while the others underwent a series of ear-trainings quite as interesting as the sight-exercises. Practically the same results were obtained upon examining the parts of the brains employed for observing sounds. Other tests with the senses of smell and taste showed the same results. And going still deeper into this mysterious science, he found that rabbits made to see only one color all their lives had brain matter in the color-seeing regions chemically different from that of other rabbits subjected solely to another shade.

One dog was made to go through certain exercises daily with his right leg, another one with his left leg. On examining their brains he found the former to have a marvellous development in what is known as the "right-leg area," while the other had the same abnormal condition of the "left-leg area." One dog was prevented from walking all during life. Another was made to follow a huckster's wagon through the streets every day. The difference in their "leg areas" was as great as that in the "color areas" of the dogs confined in the dark and those trained with the pans, etc.

But still more marvellous! Certain dogs were made to go through leg-exercises in response to certain sounds, while others did the same on being shown certain colors. In the first lot there was an unusually thick development of the fibre connecting the leg area with the hearing area, and in the latter there was the same between the leg area and the seeing area.

The fact that fibres became enlarged between areas of the brain exercised sympathetically convinces him that our memory passes from one event to another through a continuation of these fibres. Thus it would seem that these various compartments each contain an electric battery for imparting a separate action or thought, and that when several of these are used at once they develop wires connecting one with another. When our recollections, therefore, are carrying us back from one line of thought to another, these memory strings doubtless are struck in familiarly harmonious chords.

Professor Gates further believes that there is a chemical basis for both right and wrong. Good and evil thoughts produce characteristic brain structures. If his studies are applied to the fast developing medico-legal sciences, it would appear that before many years there will be little guesswork in determining the life-histories of executed criminals.

But Professor Gates would apply his new discoveries to education. He would develop brain-architects rather than brain-stuffers. Every time we indulge in certain thought or action, its corresponding section of the brain, from which it is derived, becomes more developed. Hence it is easier the next time to exert the thought or prompt the action, just as it becomes easier and easier each day to carry a heavy load. In the first instance the muscles—so to speak—of the mind are enlarged; in the second the development is in the muscles of the body. A bad habit, therefore, is mental strength developed over the wrong area. The bad "bump" works more readily than the good one, just as a muscular arm is more active and willing to strike than a weak one.

On to Buffalo!

---

The Health Department of the City of New York is now ready to supply tuberculin and mallein to the veterinary profession. They have recently issued circulars instructive of the application of these two tests, a copy of which will be useful to the daily practitioner, and which can be had for the asking.

